# Detection of neutralising antibodies to SARS-CoV-2 to determine population exposure in Scottish blood donors between March and May 2020

**DOI:** 10.2807/1560-7917.ES.2020.25.42.2000685

**Published:** 2020-10-22

**Authors:** Craig P Thompson, Nicholas E Grayson, Robert S Paton, Jai S Bolton, José Lourenço, Bridget S Penman, Lian N Lee, Valerie Odon, Juthathip Mongkolsapaya, Senthil Chinnakannan, Wanwisa Dejnirattisai, Matthew Edmans, Alex Fyfe, Carol Imlach, Kreepa Kooblall, Nicholas Lim, Chang Liu, César López-Camacho, Carol McInally, Anna L McNaughton, Narayan Ramamurthy, Jeremy Ratcliff, Piyada Supasa, Oliver Sampson, Beibei Wang, Alexander J Mentzer, Marc Turner, Malcolm G Semple, Kenneth Baillie, ISARIC4C Investigators, Heli Harvala, Gavin R Screaton, Nigel Temperton, Paul Klenerman, Lisa M Jarvis, Sunetra Gupta, Peter Simmonds, The ISARIC4C Investigators, The ISARIC4C Investigators, J Kenneth Baillie, Malcolm G Semple, Peter JM Openshaw, Gail Carson, Beatrice Alex, Benjamin Bach, Wendy S Barclay, Debby Bogaert, Meera Chand, Graham S Cooke, Annemarie B Docherty, Jake Dunning, Ana da Silva Filipe, Tom Fletcher, Christopher A Green, Ewen M Harrison, Julian A Hiscox, Antonia Ying Wai Ho, Peter W Horby, Samreen Ijaz, Saye Khoo, Paul Klenerman, Andrew Law, Wei Shen Lim, Alexander J Mentzer, Laura Merson, Alison M Meynert, Mahdad Noursadeghi, Shona C Moore, Massimo Palmarini, William A Paxton, Georgios Pollakis, Nicholas Price, Andrew Rambaut, David L Robertson, Clark D Russell, Vanessa Sancho-Shimizu, Janet T Scott, Thushan de Silva, Louise Sigfrid, Tom Solomon, Shiranee Sriskandan, David Stuart, Charlotte Summers, Richard S Tedder, Emma C Thomson, Roger Thompson AA, Ryan S Thwaites, Lance CW Turtle, Maria Zambon, Hayley Hardwick, Chloe Donohue, Ruth Lyons, Fiona Griffiths, Wilna Oosthuyzen, Lisa Norman, Riinu Pius, Tom M Drake, Cameron J Fairfield, Stephen Knight, Kenneth A Mclean, Derek Murphy, Catherine A Shaw, Jo Dalton, James Lee, Daniel Plotkin, Michelle Girvan, Egle Saviciute, Stephanie Roberts, Janet Harrison, Laura Marsh, Marie Connor, Sophie Halpin, Clare Jackson, Carrol Gamble, Gary Leeming, Andrew Law, Murray Wham, Sara Clohisey, Ross Hendry, James Scott-Brown, . William Greenhalf, Victoria Shaw, Sarah McDonald, Seán Keating, Katie A. Ahmed, Jane A Armstrong, Milton Ashworth, Innocent G Asiimwe, Siddharth Bakshi, Samantha L Barlow, Laura Booth, Benjamin Brennan, Katie Bullock, Benjamin WA Catterall, Jordan J Clark, Emily A Clarke, Sarah Cole, Louise Cooper, Helen Cox, Christopher Davis, Oslem Dincarslan, Chris Dunn, Philip Dyer, Angela Elliott, Anthony Evans, Lorna Finch, Lewis WS Fisher, Terry Foster, Isabel Garcia-Dorival, Willliam Greenhalf, Philip Gunning, Catherine Hartley, Antonia Ho, Rebecca L Jensen, Christopher B Jones, Trevor R Jones, Shadia Khandaker, Katharine King, Robyn T. Kiy, Chrysa Koukorava, Annette Lake, Suzannah Lant, Diane Latawiec, L Lavelle-Langham, Daniella Lefteri, Lauren Lett, Lucia A Livoti, Maria Mancini, Sarah McDonald, Laurence McEvoy, John McLauchlan, Soeren Metelmann, Nahida S Miah, Joanna Middleton, Joyce Mitchell, Shona C Moore, Ellen G Murphy, Rebekah Penrice-Randal, Jack Pilgrim, Tessa Prince, Will Reynolds, P. Matthew Ridley, Debby Sales, Victoria E Shaw, Rebecca K Shears, Benjamin Small, Krishanthi S Subramaniam, Agnieska Szemiel, Aislynn Taggart, Jolanta Tanianis-Hughes, Jordan Thomas, Erwan Trochu, Libby van Tonder, Eve Wilcock, J. Eunice Zhang, Kayode Adeniji, Daniel Agranoff, Ken Agwuh, Dhiraj Ail, Ana Alegria, Brian Angus, Abdul Ashish, Dougal Atkinson, Shahedal Bari, Gavin Barlow, Stella Barnass, Nicholas Barrett, Christopher Bassford, David Baxter, Michael Beadsworth, Jolanta Bernatoniene, John Berridge, Nicola Best, Pieter Bothma, David Brealey, Robin Brittain-Long, Naomi Bulteel, Tom Burden, Andrew Burtenshaw, Vikki Caruth, David Chadwick, Duncan Chambler, Nigel Chee, Jenny Child, Srikanth Chukkambotla, Tom Clark, Paul Collini, Catherine Cosgrove, Jason Cupitt, Maria-Teresa Cutino-Moguel, Paul Dark, Chris Dawson, Samir Dervisevic, Phil Donnison, Sam Douthwaite, Ingrid DuRand, Ahilanadan Dushianthan, Tristan Dyer, Cariad Evans, Chi Eziefula, Chrisopher Fegan, Adam Finn, Duncan Fullerton, Sanjeev Garg, Sanjeev Garg, Atul Garg, Effrossyni Gkrania-Klotsas, Jo Godden, Arthur Goldsmith, Clive Graham, Elaine Hardy, Stuart Hartshorn, Daniel Harvey, Peter Havalda, Daniel B Hawcutt, Maria Hobrok, Luke Hodgson, Anil Hormis, Michael Jacobs, Susan Jain, Paul Jennings, Agilan Kaliappan, Vidya Kasipandian, Stephen Kegg, Michael Kelsey, Jason Kendall, Caroline Kerrison, Ian Kerslake, Oliver Koch, Gouri Koduri, George Koshy, Shondipon Laha, Steven Laird, Susan Larkin, Tamas Leiner, Patrick Lillie, James Limb, Vanessa Linnett, Jeff Little, Michael MacMahon, Emily MacNaughton, Ravish Mankregod, Huw Masson, Elijah Matovu, Katherine McCullough, Ruth McEwen, Manjula Meda, Gary Mills, Jane Minton, Mariyam Mirfenderesky, Kavya Mohandas, Quen Mok, James Moon, Elinoor Moore, Patrick Morgan, Craig Morris, Katherine Mortimore, Samuel Moses, Mbiye Mpenge, Rohinton Mulla, Michael Murphy, Megan Nagel, Thapas Nagarajan, Mark Nelson, Igor Otahal, Mark Pais, Selva Panchatsharam, Hassan Paraiso, Brij Patel, Natalie Pattison, Justin Pepperell, Mark Peters, Mandeep Phull, Stefania Pintus, Jagtur Singh Pooni, Frank Post, David Price, Rachel Prout, Nikolas Rae, Henrik Reschreiter, Tim Reynolds, Neil Richardson, Mark Roberts, Devender Roberts, Alistair Rose, Guy Rousseau, Brendan Ryan, Taranprit Saluja, Aarti Shah, Prad Shanmuga, Anil Sharma, Anna Shawcross, Jeremy Sizer, Manu Shankar-Hari, Richard Smith, Catherine Snelson, Nick Spittle, Nikki Staines, Tom Stambach, Richard Stewart, Pradeep Subudhi, Tamas Szakmany, Kate Tatham, Jo Thomas, Chris Thompson, Robert Thompson, Ascanio Tridente, Darell Tupper-Carey, Mary Twagira, Andrew Ustianowski, Nick Vallotton, Lisa Vincent-Smith, Shico Visuvanathan, Alan Vuylsteke, Sam Waddy, Rachel Wake, Andrew Walden, Ingeborg Welters, Tony Whitehouse, Paul Whittaker, Ashley Whittington, Meme Wijesinghe, Martin Williams, Lawrence Wilson, Sarah Wilson, Stephen Winchester, Martin Wiselka, Adam Wolverson, Daniel G Wooton, Andrew Workman, Bryan Yates, Peter Young

**Affiliations:** 1 Department of Zoology, University of Oxford, Oxford, United Kingdom; 2 Peter Medawar Building for Pathogen Research, Oxford, United Kingdom; 3 Department of Paediatric Medicine, University of Oxford, University of Oxford, Oxford, United Kingdom; 4 Nuffield Department of Medicine, University of Oxford, Oxford, United Kingdom; 5 Zeeman Institute for Systems Biology and Infectious Disease Epidemiology Research, School of Life Sciences, The University of Warwick, Coventry, United Kingdom; 6 Wellcome Centre for Human Genetics, Nuffield Department of Medicine, University of Oxford, Oxford, United Kingdom; 7 National Microbiology Reference Unit, Scottish National Blood Transfusion Service, Edinburgh, United Kingdom; 8 Oxford Centre for Diabetes, Endocrinology and Metabolism, Churchill Hospital, University of Oxford, Oxford, United Kingdom; 9 Wellcome Centre for Human Genetics, University of Oxford, Roosevelt Drive, Oxford, United Kingdom; 10 Health Protection Unit in Emerging and Zoonotic Infection, Faculty of Health and Life Sciences, University of Liverpool, Liverpool, United Kingdom; 11 NIHR Health Protection Research Unit in Emerging and Zoonotic Infections, Faculty of Health and Life Sciences, University of Liverpool, Liverpool, United Kingdom; 12 The members of the ISARIC4C Investigators are listed under the investigator tab; 13 Infection and Immunity, University College London, London, United Kingdom; 14 Viral Pseudotype Unit, Medway School of Pharmacy, University of Kent, Chatham, United Kingdom

**Keywords:** COVID19, SARS-CoV-2, pandemic, serology, surveillance, Scotland

## Abstract

**Background:**

The progression and geographical distribution of severe acute respiratory syndrome coronavirus 2 (SARS-CoV-2) infection in the United Kingdom (UK) and elsewhere is unknown because typically only symptomatic individuals are diagnosed. We performed a serological study of blood donors in Scotland in the spring of 2020 to detect neutralising antibodies to SARS-CoV-2 as a marker of past infection and epidemic progression.

**Aim:**

Our objective was to determine if sera from blood bank donors can be used to track the emergence and progression of the SARS-CoV-2 epidemic.

**Methods:**

A pseudotyped SARS-CoV-2 virus microneutralisation assay was used to detect neutralising antibodies to SARS-CoV-2. The study comprised samples from 3,500 blood donors collected in Scotland between 17 March and 18 May 2020. Controls were collected from 100 donors in Scotland during 2019.

**Results:**

All samples collected on 17 March 2020 (n = 500) were negative in the pseudotyped SARS-CoV-2 virus microneutralisation assay. Neutralising antibodies were detected in six of 500 donors from 23 to 26 March. The number of samples containing neutralising antibodies did not significantly rise after 5–6 April until the end of the study on 18 May. We found that infections were concentrated in certain postcodes, indicating that outbreaks of infection were extremely localised. In contrast, other areas remained comparatively untouched by the epidemic.

**Conclusion:**

Although blood donors are not representative of the overall population, we demonstrated that serosurveys of blood banks can serve as a useful tool for tracking the emergence and progression of an epidemic such as the SARS-CoV-2 outbreak.

## Introduction

Severe acute respiratory syndrome coronavirus 2 (SARS-CoV-2) emerged in late 2019 in Hubei province, China as a cause of respiratory disease occasionally leading to coronavirus disease (COVID-19) [1,2]. Older age, male sex, smoking and comorbidities such as cardiac disease, hypertension and diabetes have been identified as risk factors for severe infections [3,4].

Symptomatic individuals typically exhibit fever, cough and shortness of breath 2–14 days after infection [5]. However, an unknown proportion of individuals experience no symptoms [6-8]. Antibody responses in both symptomatic and asymptomatic individuals are detectable in the blood 14–28 days after infection [9,10]. Subsequently, antibody levels drop and can become undetectable by some antibody assays in the early convalescent phase [9,11,12].

In this study, we used blood donors as a means of estimating population exposure from the start of the pandemic in March through to mid-May when PCR-detected cases in the United Kingdom (UK) had plateaued [13,14]. The detection frequency of neutralising antibodies in blood donors and a discussion of its applicability for estimating population level exposure are presented.

## Methods

### Samples

We analysed six batches of 500 plasma samples collected on 17 March, 21–23 March, 5–6 April, 18–20 April, 2–4 May and 16–18 May from Scotland. Each batch was sampled from a range of health boards across Scotland, with the coverage varying between batches. An additional 500 samples from the Greater Glasgow region, collected between 2 and 4 May were also analysed. This yielded a total of 3,500 post-pandemic blood donor samples. Of these samples, 53.4% were from female donors. The median age of donors was 47 years (IQR: 34–56); children under 16 years are not permitted to donate blood. As negative controls, we tested in parallel 100 blood donor samples from the Scottish National Blood Transfusion Service (SNBTS) anonymous archive collected between September 2018 and December 2019 (IRAS project number 18005), before the first reports of the spread of SARS-CoV-2 in China [1,2]. Seventeen control samples from contract-traced individuals who were PCR-confirmed as SARS-CoV-2 infected were used as positive controls in the study. All the individuals from whom the positive control sera samples were taken had asymptomatic SARS-CoV-2 infections and were recruited through the International Severe Acute Respiratory and Emerging Infection Consortium (ISARIC) World Health Organization Clinical Characterisation Protocol UK (CCP-UK) at the time point of discharge plus 28 days. Samples were heat-inactivated before serological testing by incubation at 56 °C for 30 min.

### SARS-CoV-2 pseudotype microneutralisation assay

A lentivirus-based SARS-CoV-2 pseudovirus particle was constructed displaying the full spike protein on the surface of the pseudotyped virus using a synthetic codon-optimised SARS-CoV-2 expression construct (NCBI reference sequence: YP_009724390.1). Virus infectivity was determined by titration on HEK 293T ACE2-plasmid-transfected cells as previously described [15]. Neutralisation titres are expressed as 50% inhibitory concentration (IC_50_) values. During the assay, plates were barcoded and controls were periodically added to the runs. Laboratory staff were blinded regarding the arrangement of positive controls periodically added to the assay plates.

### Titration

Pre-pandemic samples and samples collected on 17 March and 21–23 March were all titrated to optimise the neutralisation assay. After this point, samples were initially screened for neutralisation using the highest 1:20 dilution. Dilutions of 1:20 were performed in triplicate along with virus only, no virus and positive control wells. Samples that produced a mean RLU two standard deviations below the mean RLU of all the samples on the plate were then titrated out to obtain IC_50_ values.

### Enzyme-linked immunosorbent assay

Antibodies to the trimeric spike protein were detected by ELISA. MAXISORP immunoplates (442404; NUNC; Merck, Darmstadt, Germany) were coated with StrepMAB-Classic (2–1507–001; IBA Life Sciences, Göttingen, Germany). Plates were blocked with 2% skimmed milk in phosphate buffered saline (PBS) for 1 h and then incubated with 0.125 μg of soluble SARS-CoV-2 trimeric spike protein or 2% skimmed milk in PBS. After 1 h, plasma was added at 1:50 dilution, followed by alkaline phosphatase (AP)-conjugated anti-human IgG (A9544; Merck, Darmstadt, Germany) at 1:10,000 dilution or AP-conjugated anti-human IgM (A9794; Merck, Darmstadt, Germany) at 1:5,000 dilution. The reaction was developed by the addition of p-nitrophenyl phosphate (PNPP, Merck, Darmstadt, Germany) substrate and stopped with NaOH. The absorbance was measured at 405 nm after 1 h. Further information is provided in Adams et al. [16].

### Estimating the 50% inhibitory concentration

The RLU for each well were standardised against technical positive (cells and virus without serum) and negative (cells without serum or virus) controls on each plate to determine a percentage neutralisation value. We calculated an average neutralisation across the two sample replicates on each plate (for each dilution). Dilution curves were fit to each sample, with the percentage neutralisation modelled as a logistic function of the dilution factor. This yielded an IC_50_ value for each sample where a curve could be fit; samples that showed no dilution response because of complete or no neutralisation were not given an IC_50_ value. Dilution curves were estimated using nonlinear least squares in R version 3.6.3 [17]. An error-weighted mean of the IC_50_ value was calculated for samples that were repeated on more than one plate. We classified positive samples as having an IC_50_ value greater than the largest negative control (1:69) with a standard error less than or equal to the least neutralising positive control.

### Determining test sensitivity and specificity

Test sensitivity (probability of neutralisation in a given positive serum) and specificity (probability of a negative result given no exposure) was estimated using 17 (RT-PCR-confirmed) positive controls and 100 pre-pandemic blood donor samples as negative controls. The highest IC_50_ observed for a negative control was used as a threshold to determine positive samples (giving 100% specificity; 95% credible interval (CI): 98.10–100; n = 117). Of the 17 positive controls, 16 samples neutralised with high confidence, giving an estimated sensitivity of 94.11% (95% CI: 79.17–99.98).

### Accounting for sensitivity and specificity in sample prevalence estimates

Uncertainty in test sensitivity and specificity can be propagated to sample prevalence estimates using a simple hierarchical Bayesian model [18]. The number of positive tests in the positive (n^+^ = 16) and negative (n^−^ = 0) control groups was modelled as a binomial distribution:

$$n^{+}∼Binom\left(\pi^{+},\;N^{+}\right)$$

$$n^{-}∼Binom\left(1-\pi^{-},\;N^{-}\right)$$

where the sensitivity is given by π^+^ and the specificity by π^−^ (*N*^+^ = 17 and *N*^−^ = 100 are the number of positive and negative controls, respectively). An estimate of the true proportion of positive sera for samples from a given week and health board (*p_w,h_*) comprises neutralising sera that were missed ([1 − π^+^]) and those incorrectly identified as neutralising samples (from [1 − π^−^]). The observed number of positive samples for the week w and health board h (n_w,h_) was modelled as a binomial distribution accounting for test performance:

$$n_{w,h}∼Binom\left(p_{w,h}\;\pi^{+}+\left[1-p_{w,h}\right]\left[1-\pi^{-}\right],\;{\;N}_{w,h}\right)$$

with *N_w,h_* the number of samples from each health board in each week. Using this method, the uncertainty in test specificity and sensitivity is propagated to the estimate of the seroprevalence; this results in broader credible intervals that better reflect the inherent uncertainty in test parameters.

### Modelling sample prevalence

In estimating seroprevalence, we assumed that neutralising antibodies did not wane in the blood donor population during the survey period and accrued to an equilibrium [12]. Making this assumption, we can fit the logistic function to the time series of sample seroprevalence:

$$p_{w,h}=\frac{\theta_{h}}{\left(1\;+\;exp\left[-\rho_{h}\;\left(w\;-\;\tau_{h}\right)\right]\right)}$$

$$\theta_{h}\sim \beta \left(\phi_{\theta },\eta_{\theta }\right)$$

$$\rho_{h}\sim N\left(\mu_{\rho },{\sigma_{\rho }}^{2}\right)$$

$$\tau_{h}\sim N\left(\mu_{\tau },{\sigma_{\tau }}^{2}\right)$$

Here, θ*_h_* is the equilibrium seroprevalence, ρ*_h_* is the rate with which the seroprevalence approaches this maximum and τ*_h_* is the midpoint of the logistic curve for each health board. Parameters were modelled using hierarchical distributions across health boards (the maximum as a beta to bound it between 0 and 1, the rate and the midpoint as a normal distribution). Priors are given in the Supplementary material. The model was fit in R version 3.6.3 using the Bayesian inference package JAGS version 4.3.0 [19]. Models were run across six chains until convergence (potential scale reduction factor less than 1.02 and effective sample size > 10,000).

### Ethical statement

Ethical approval was obtained for the SNBTS anonymous archive - IRAS project number 18005. SNBTS blood donors gave fully informed consent to virological testing, donation was made under the SNBTS Blood Establishment Authorisation and the study was approved by the SNBTS Research and Sample Governance Committee.

## Results

**Figure 1:**
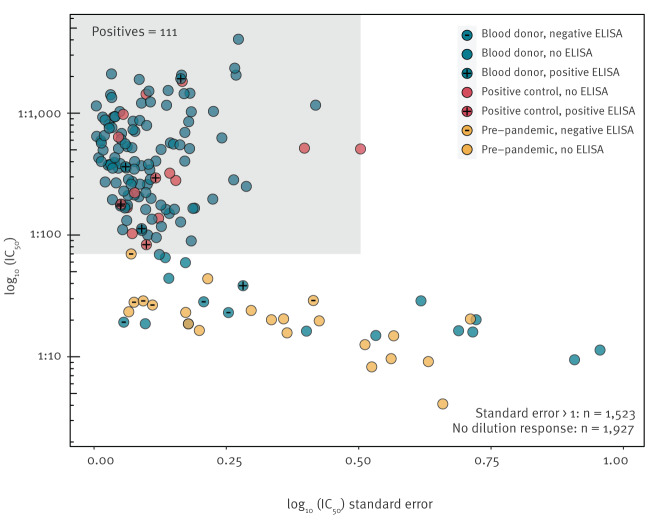
Selection criteria for classifying a sample as SARS-CoV-2-neutralising, Scotland, March–May 2020 (n = 3,617)

ELISA: enzyme-linked immunosorbent assay; IC_50_: 50% inhibitory concentration; SARS-CoV-2: severe acute respiratory syndrome coronavirus 2.

The estimated IC_50_ values and standard errors for the control and blood donor samples are shown in Figure 1. Of the 3,500 post-pandemic blood donor samples, a total of 111 contained anti-SARS-CoV-2 neutralising antibodies using the IC_50_ and standard error-based thresholds described in the Methods. The results of the neutralisation assay were positively correlated with ELISA optical density (Supplementary Figure S2; Pearson’s correlation coefficient = 0.86; p < 0.001).

Samples are required to have an estimated IC_50_ and a standard error at least as small as the worst-neutralising positive control. This penalises samples with poorly defined inhibitory concentrations. Pre-pandemic samples are shown in yellow, positive controls in red and blood donor samples in blue. ELISA results are annotated as + or − on the points.

**Figure 2:**
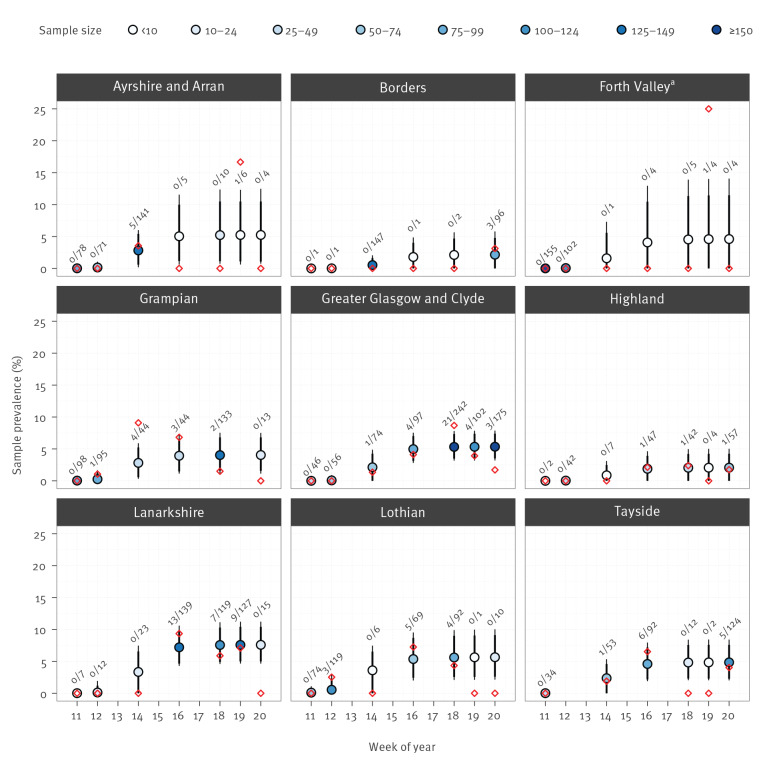
SARS-CoV-2 antibody prevalence estimates for each health board through time using the model outlined in the methods, blood donors, Scotland, March–May 2020 (n = 3,500)

^a^ Poor sample coverage and single positive result for the estimates for Forth Valley could be fit by a range of values.

No samples from 17 March showed neutralising activity. Blood donor samples obtained from donations during 21–23 March, 5–6 April, 18–20 April, 2–4 May and 16–18 May contained neutralising anti-SARS-CoV-2 antibodies (Figure 2). The number of samples containing neutralising antibodies did not rise significantly after 5–6 April.

Filled circles: median prevalence estimates, colour denoting sample size; thick and thin error bars: 90 and 95% highest density intervals, respectively. Red diamonds: raw percentage estimates from the data.

**Figure 3:**
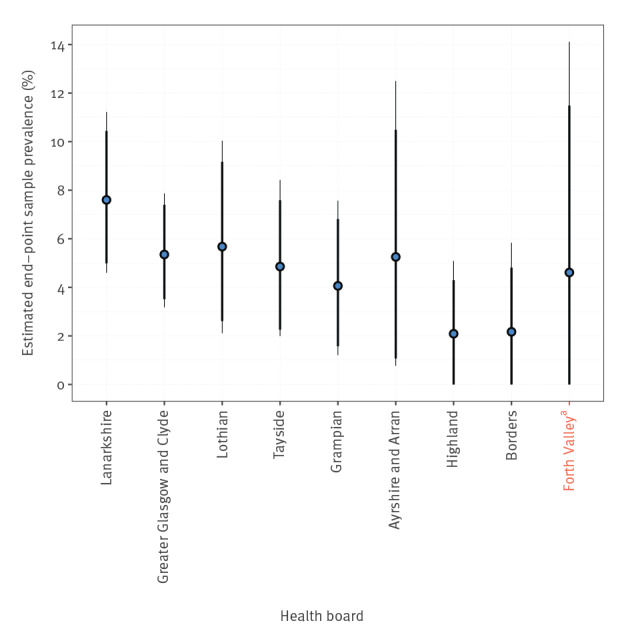
Estimates of SARS-CoV-2 antibody prevalence at the end of our study period (the parameter θ*_h_* from the logistic equation), ordered by the lower 95% highest density interval, blood donors, Scotland March–May 2020 (n = 3,500)

**Figure 4:**
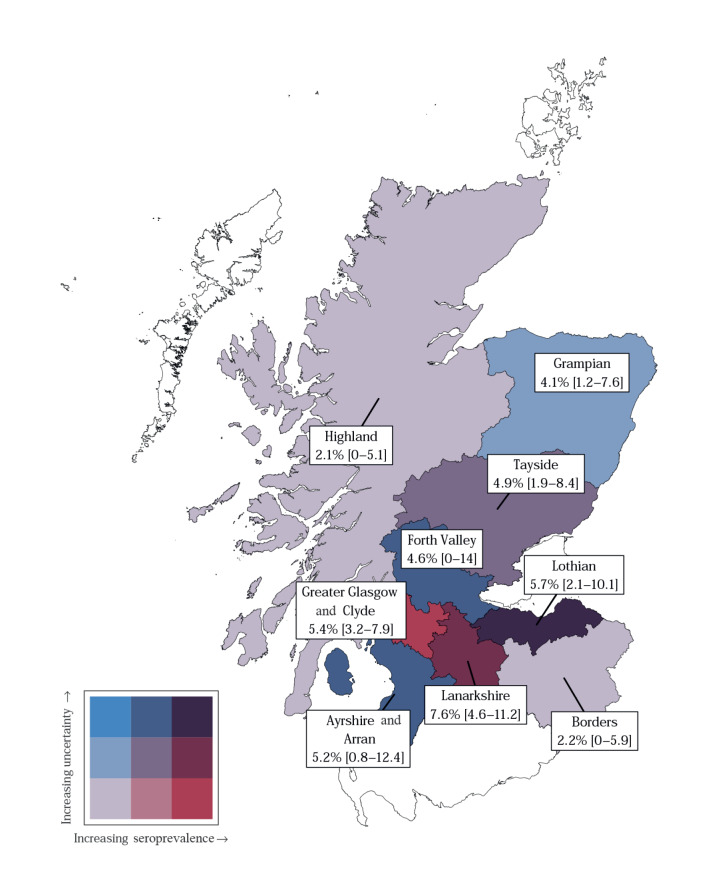
Health boards showing estimated endpoint seroprevalences for SARS-CoV-2 in blood donors, Scotland, March–May 2020 (n = 3,500)

Estimates of seroprevalence in the healthcare boards, based on the final sampling point between the 16–18 May, are illustrated in Figures 3 and 4. The lowest uncertainty was associated with estimates from the Greater Glasgow and Clyde health board (5.35%; 95% highest density interval (HDI): 3.19–7.89); Tayside, Lothian and Grampian had similar median estimates with higher uncertainty. Lanarkshire was predicted to have the highest seroprevalence of all health boards (7.59%; 95% HDI: 4.60–11.20) while the Highlands and Borders had the lowest seroprevalence of around 2.08 (95% HDI: 0–5.08) and 2.16 (95% HDI: 0–5.85), respectively. Throughout this period, IC_50_ values did not show a statistically significant difference between weeks (Supplementary Figure S3). No statistically significant variation in IC_50_ value was seen based on age or sex (Supplementary Figure S4).

Blue points: median parameter estimate; thick and thin error bars: 90 and 95% highest density intervals, respectively. All sample estimates are characterised by high uncertainty: the Lanarkshire health board was predicted to have the highest prevalence; Greater Glasgow and Clyde were estimated with the most confidence, while Forth Valley, Borders and Highland could not be interpreted and estimates for the Forth Valley should be treated with scepticism because of poor sampling (see Figure 2).

^a^ Poor sample coverage and single positive result for the estimates for Forth Valley could be fit by a range of values.

White health boards were not sufficiently sampled to generate estimates. The colour scale denotes the confidence and seroprevalence in each health board. Labels give estimated blood donor seroprevalence for the health board with associated 95% highest density intervals.

**Figure 5:**
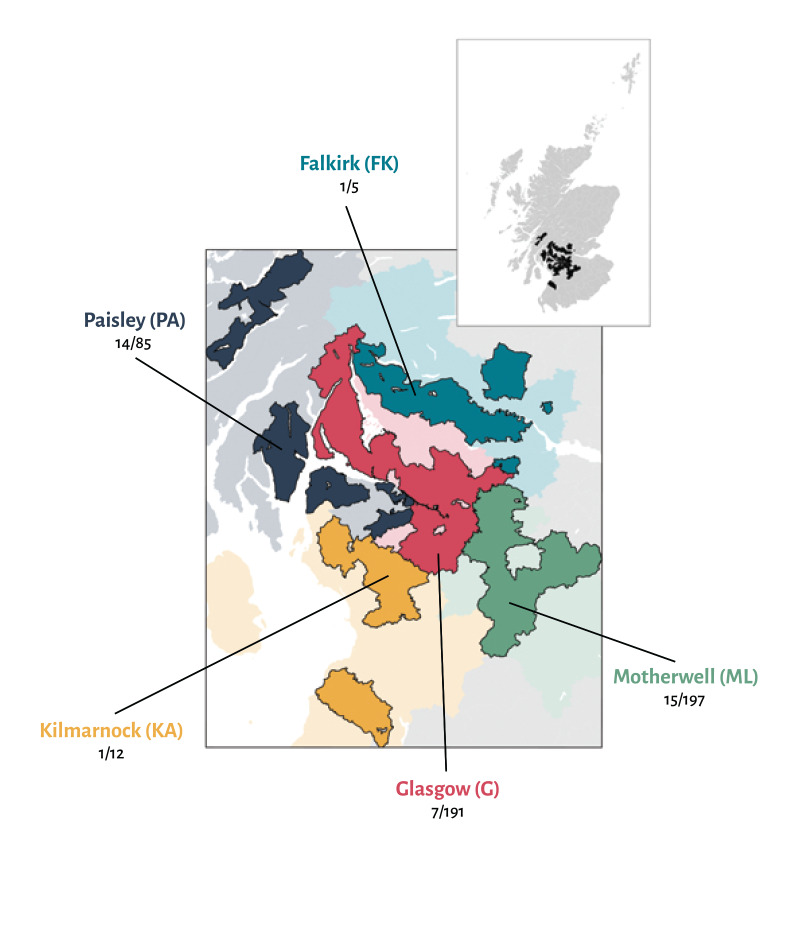
Raw counts of positive SARS-CoV-2 samples in the additional survey of postcodes close to Glasgow, blood donors, March–May 2020 (n = 490)

The outbreaks as a whole in Scotland were concentrated in the major urban centres, Glasgow and Edinburgh, and the Lanarkshire health authority region (Figures 3 and 4). To explore this phenomenon further, we performed a separate analysis of 490 samples from the Greater Glasgow region collected between 18 and 20 April. Of these 490 samples, 42 had neutralising antibodies. Analysis of the distribution of samples containing neutralising antibodies by postcodes showed that most of these samples located in the Paisley (14/85) and Motherwell (15/197) postcodes of Greater Glasgow, indicating that outbreaks in the city and its surrounding localities are localised. By comparison, Central Glasgow had comparatively few samples containing neutralising antibodies (7/195; Figure 5).

490 samples fell within districts adjacent to the city.

## Discussion

Our study strengthens existing evidence that blood donors can be used as a sentinel population to track the emergence and progression of an epidemic.

While the demographics of blood donors differ in several aspects from the general population, most notably because of the exclusion of those at risk for blood-borne viruses (HIV, hepatitis B and C virus) and syphilis, they might be considered a reasonable representation of the adult population in the absence of any obvious predisposing factors for infection. The only other general exclusions were a 4-week and a 4-month donation deferral period in those people who travelled to specified countries at risk for arbovirus and malaria infections, respectively.

However, estimates of seroprevalence are complicated by non-uniform sampling. The blood donations collected and tested in this study were focused in specific postcodes, based on the locations where weekly donations took place. This produces an added level of complexity as our data showed that outbreaks are focused in specific communities even on the scale of a medium-sized city such as Glasgow. This is further confounded by the absence of samples from individuals younger than 18 and older than 75 years.

The results presented in this study are based on a formally not validated assay. However, by using contact-traced asymptomatic individuals who had been PCR-confirmed as infected by SARS-CoV-2 and 100 blood donations obtained before the epidemic, we were able to ascertain the sensitivity of the assay. Furthermore, a second ELISA was used to confirm the analysis. As this assay detected 16 of 17 PCR-confirmed asymptomatic cases, we estimated its sensitivity at 94.11% (95% CI: 79.17–99.98). Other studies have previously shown that the pMN assay correlates well with other laboratory-based and commercial serological assays [20].

Our assay is designed to be specific for SARS-CoV-2. There are four seasonal coronaviruses, HKU1, OC43, NL63 and 229E, which circulate during the winter months [21]. The 100 pre-pandemic samples collected in the winter months of 2019 will have been from donors previously infected with seasonal coronaviruses (but not SARS-CoV-2). By setting the cut-off for our assay above the highest IC_50_ value observed in the 100 pre-pandemic samples, we ensured the specificity of the assay and can have a high degree of confidence that the antibodies detected in the samples from March to May 2020 were generated by SARS-CoV-2 infection. The utility of using pMN assays and ELISA to track population exposure is dependent on the assumptions (i) that every infected individual seroconverts and (ii) that once seroconverted, the antibodies remain circulating in the blood at detectable levels. A decrease in total antibody and neutralising antibody titres has been noted in samples drawn up to 2 months after the peak neutralising antibody response (ca 3–4 weeks after infection). In some instances, antibody levels become undetectable when tested with a specific assay and analysis methodology [9,12]. This drop in titres may lead to false negatives in the later time points. However, the dates of collection used in this study all fell within 3 months of the diagnosis of the first confirmed case in Scotland on 1 March [22]. For this reason, it is unlikely that this study is hampered by a drop in neutralising antibody levels, although future seroprevalence studies may potentially underestimate the true level of population exposure. In addition, some individuals may not seroconvert, representing a small pool of false negative patients [11].

## Conclusion

Samples containing anti-SARS-CoV-2 neutralising antibodies were detected in blood donors who gave blood between 16 and 17 March 2020 in all Scottish health boards. Subsequently, samples containing anti-SARS-CoV-2 neutralising antibodies were detected at every further time point assayed until the end of the study. Consequently, considering the 14–28 day incubation period before seroconversion, it is likely that SARS-CoV-2 began circulating in Scotland in late February 2020 and potentially earlier.

## Supplementary Data

2228000 Supplement
