# Supplementary material for: Detection of neutralising antibodies to SARS-CoV-2 to determine population exposure in Scottish blood donors between March and May 2020
Source: Euro Surveill. 2020 Oct 22;25(42):2000685. doi: 10.2807/1560-7917.ES.2020.25.42.2000685 (PMC7651873; doi:10.2807/1560-7917.ES.2020.25.42.2000685)
Supplement: Supplement [file 20-00685_THOMPSON_Supplement.pdf]

Supplementary material – additional information regarding the 'Detection of neutralising antibodies to SARS coronavirus 2 to determine population exposure in Scottish blood donors between March and May 2020'

This supplementary material is hosted by *Eurosurveillance* as supporting information alongside the article *Detection of neutralising antibodies to SARS-CoV-2 to determine population exposure in Scottish blood donors between March and May 2020*, on behalf of the authors, who remain responsible for the accuracy and appropriateness of the content. The same standards for ethics, copyright, attributions and permissions as for the article apply. Supplements are not edited by *Eurosurveillance* and the journal is not responsible for the maintenance of any links or email addresses provided therein.

Tables

parameter	*description*	*prior*	*justification*
ϕ_θ	First shape parameter for the beta distribution describing the maximum seroprevalences in each health board.	$\Gamma(0.04, 0.04)$	Equivalent to a mode of 1 and standard deviation of 5.
η_θ	Second shape parameter for the beta.	$\Gamma(4, 0.4)$	Equivalent to a mode of 10 and standard deviation of 5. In combination with the above, these priors gave weight to lower seroprevalences without being too prescriptive.
μ_ρ	Mean of the normally distributed rates of increase for the seroprevalence curves.	$\mathcal{N}(1, 1)$	Equivalent to a mean of 1 and standard deviation of 1. This covers a realistic range of gradients of increasing seroprevalence.
σ_ρ	Standard deviation for the above.	$\Gamma(1, 1)$	Equivalent to a mean of 1 and standard deviation of 1. This gave enough flexibility when estimating rates while preventing the model from exploring infeasibly steep slopes.
μ_τ	Mean of the normal distribution describing the midpoints of seroprevalence accumulation. Expressed as the number of weeks past the start of the survey.	$\mathcal{N}(4, 1)$	A mean of 4 with a standard deviation of 1. This allowed the model to explore reasonable midpoints for seroprevalence saturation with a bias towards earlier weeks of the survey.
σ_τ	Standard deviation of the logistic curve midpoints.	$\Gamma(1, 1)$	Equivalent to a mean of 1 and standard deviation of 1.

Table S1. Model priors.

Figures

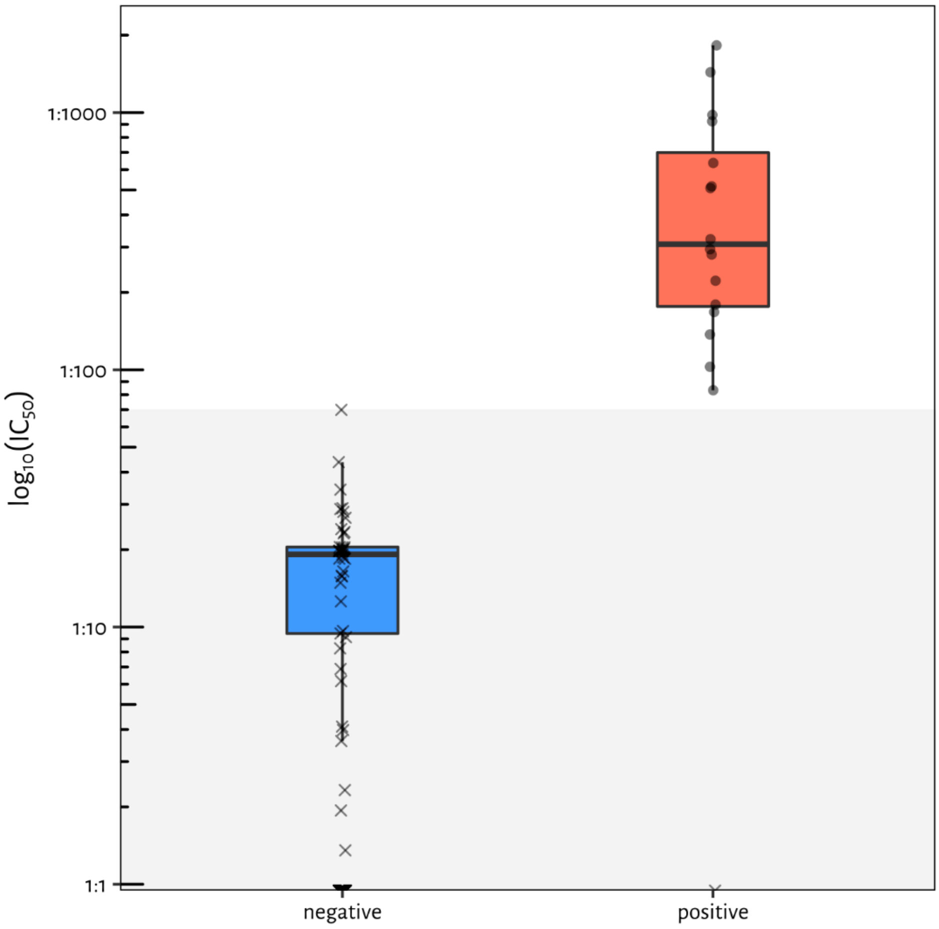

Figure S1. Inhibitory concentrations for the positive (17 RT-PCR confirmed) and negative (100 pre-pandemic blood donors) controls. Crosses indicate a sample classified as negative, circles as positive. A sample had to have an IC_{50} and a standard error at least as small as the worst neutralising negative control.

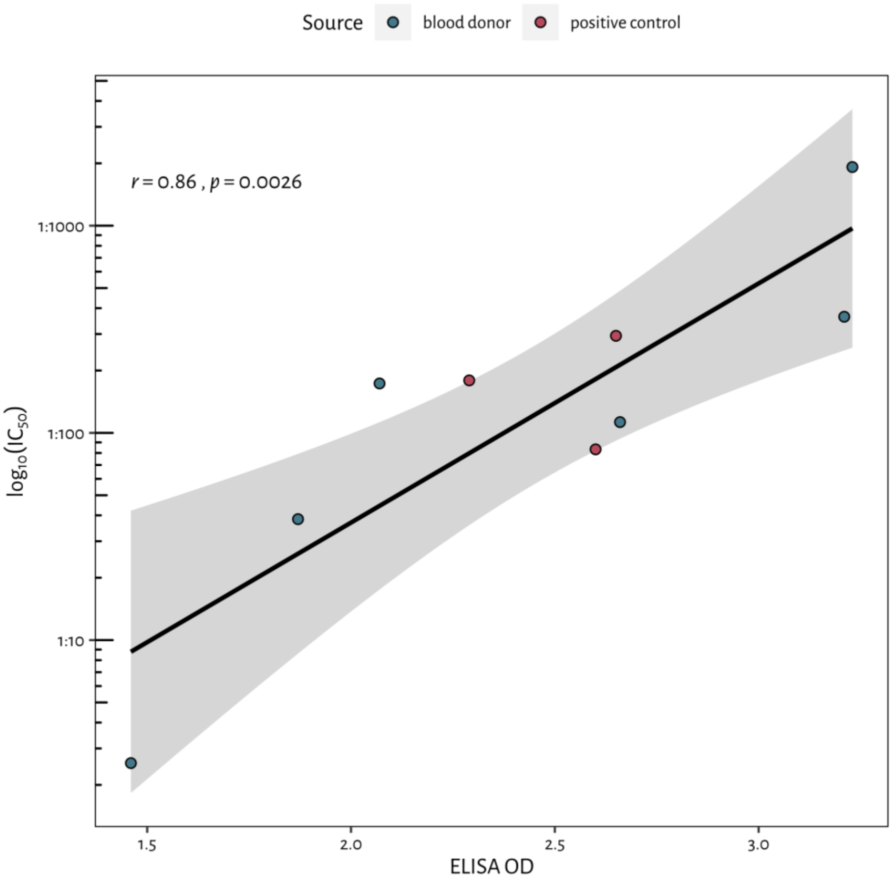

Figure S2. Correlation between inhibitory concentrations (IC50) from our neutralisation assay and the ELISA optical density. The assays were positively correlated (Pearson's correlation coefficient = 0.86, $p < 0.001$)

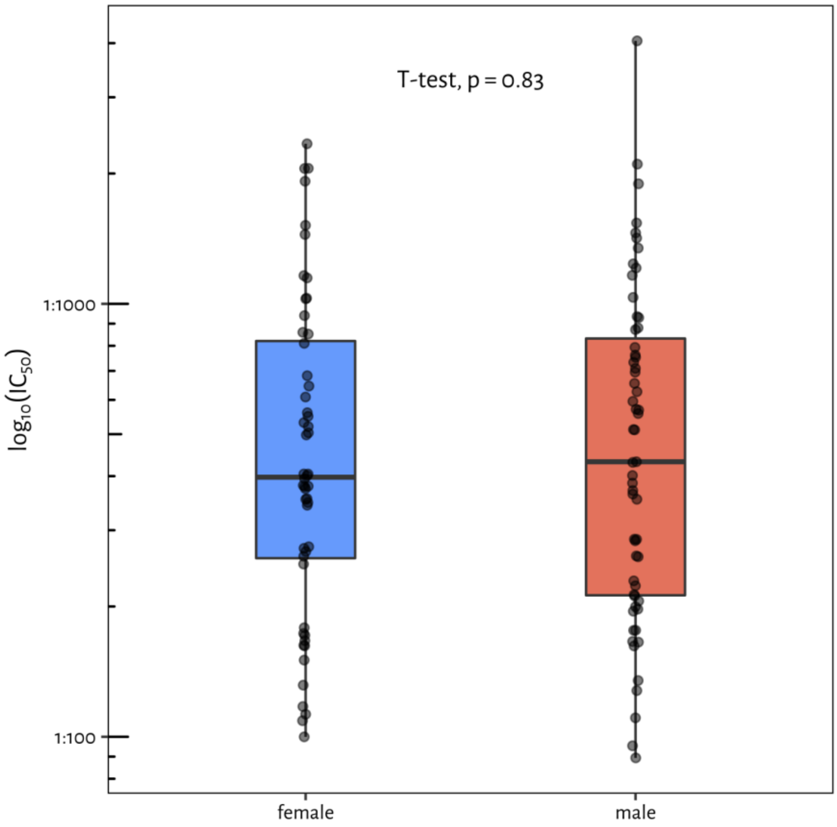

Figure S3. Comparison of 50% inhibitory concentrations (IC50) for each sex. No significant difference was found (t = -0.21, df = 107.93, p-value = 0.83).

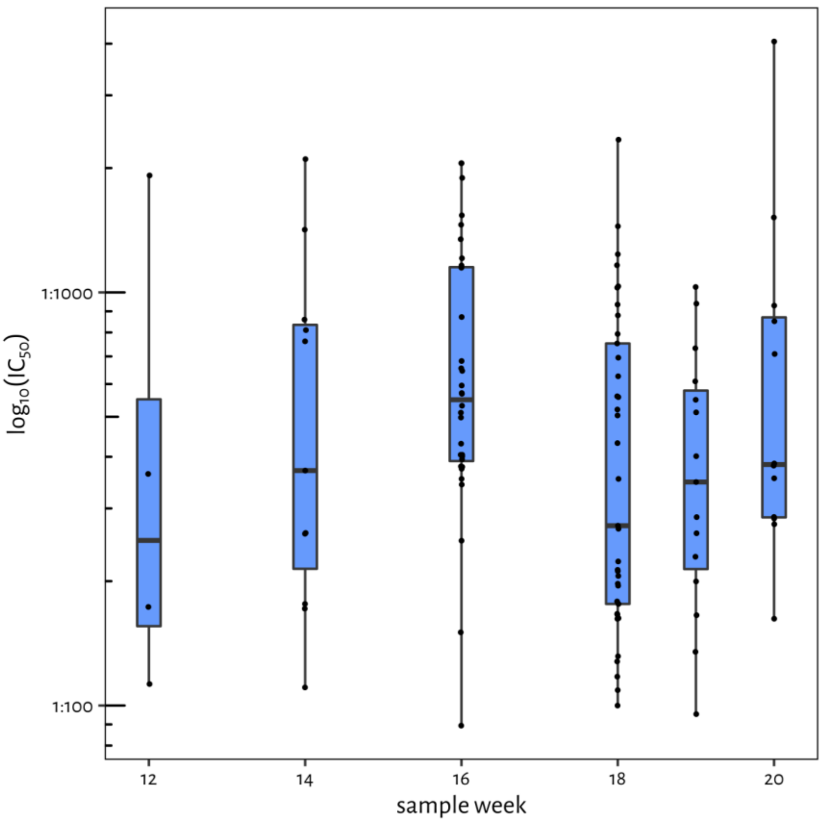

Figure S4. Comparison of 50% inhibitory concentrations (IC50) for each study week.

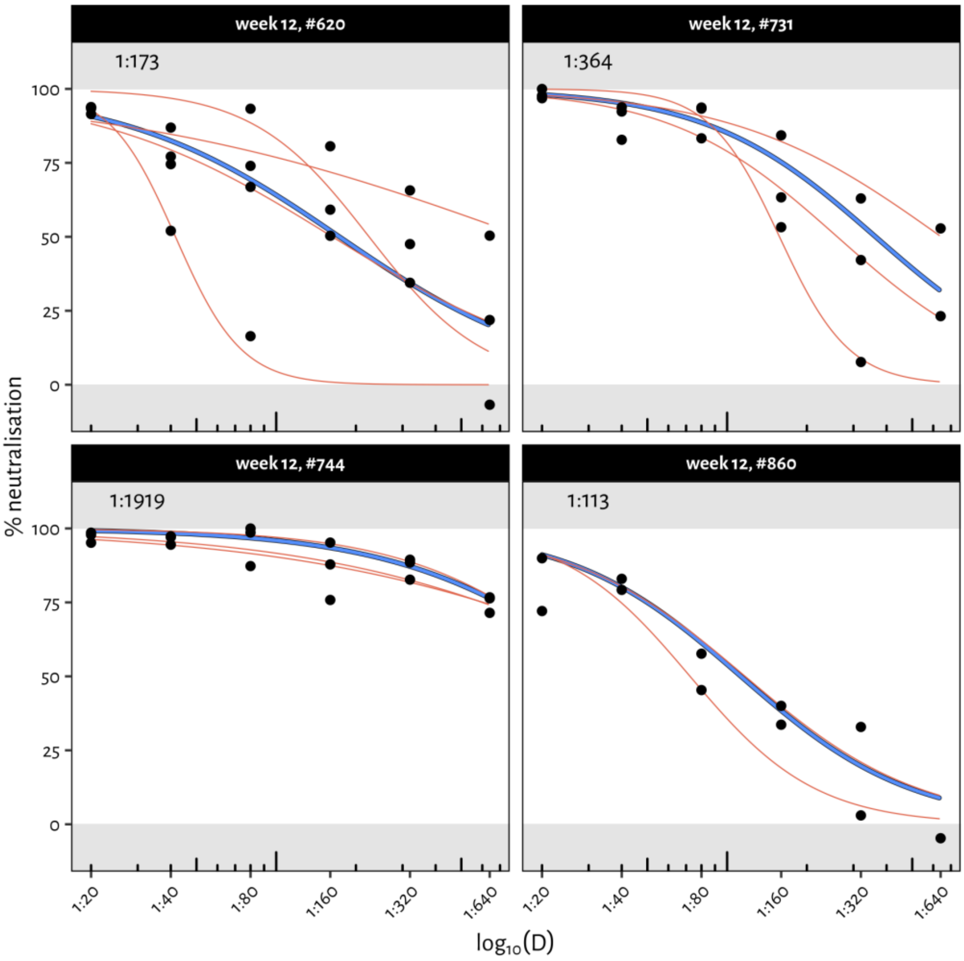

Figure S5. Dilution curves for neutralising samples from week 12. Fits for each replicate are shown in red, with the weighted mean given in blue.

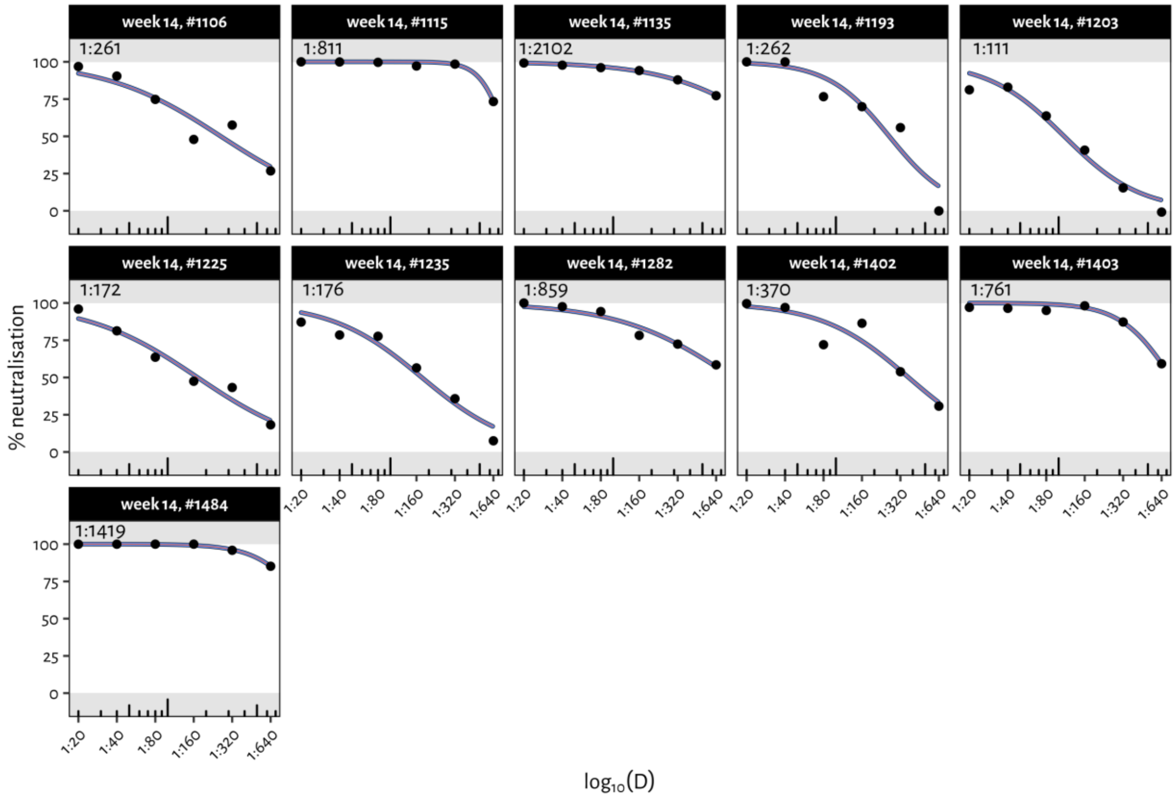

Figure S6. Dilution curves for neutralising samples from week 14. Fits for each replicate are shown in red, with the weighted mean given in blue.

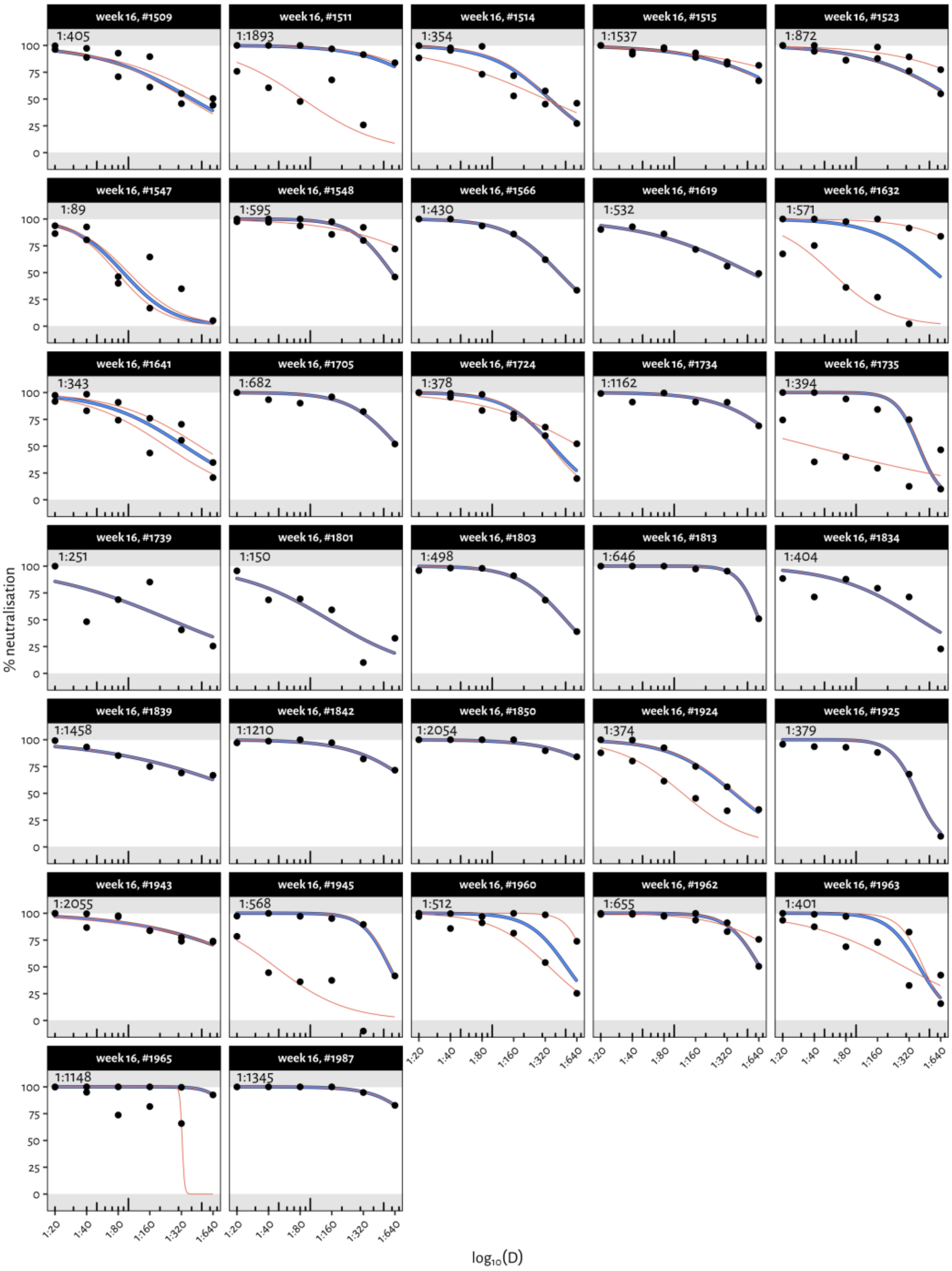

Figure S7. Dilution curves for neutralising samples from week 16. Fits for each replicate are shown in red, with the weighted mean given in blue.

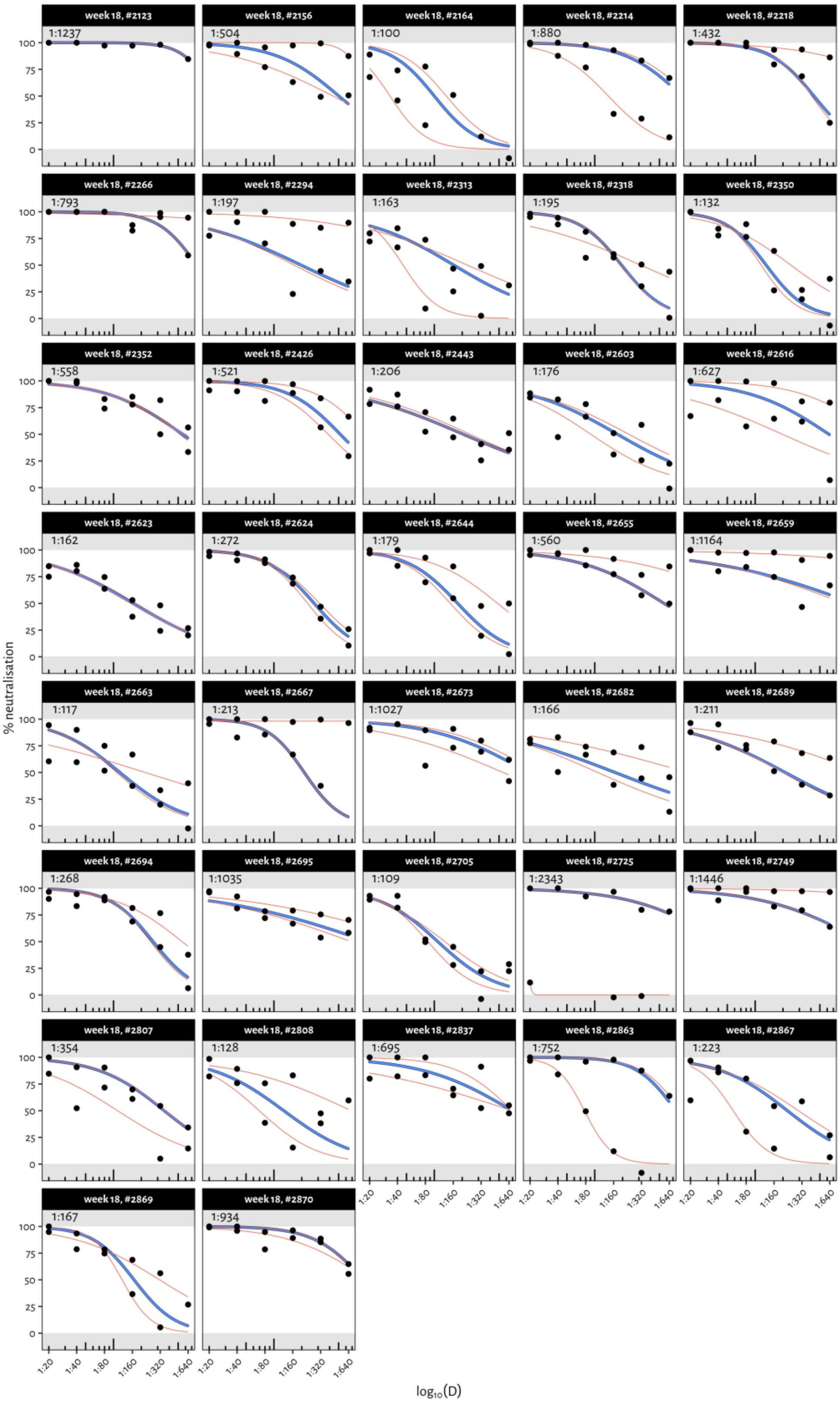

Figure S8. Dilution curves for neutralising samples from week 18. Fits for each replicate are shown in red, with the weighted mean given in blue.

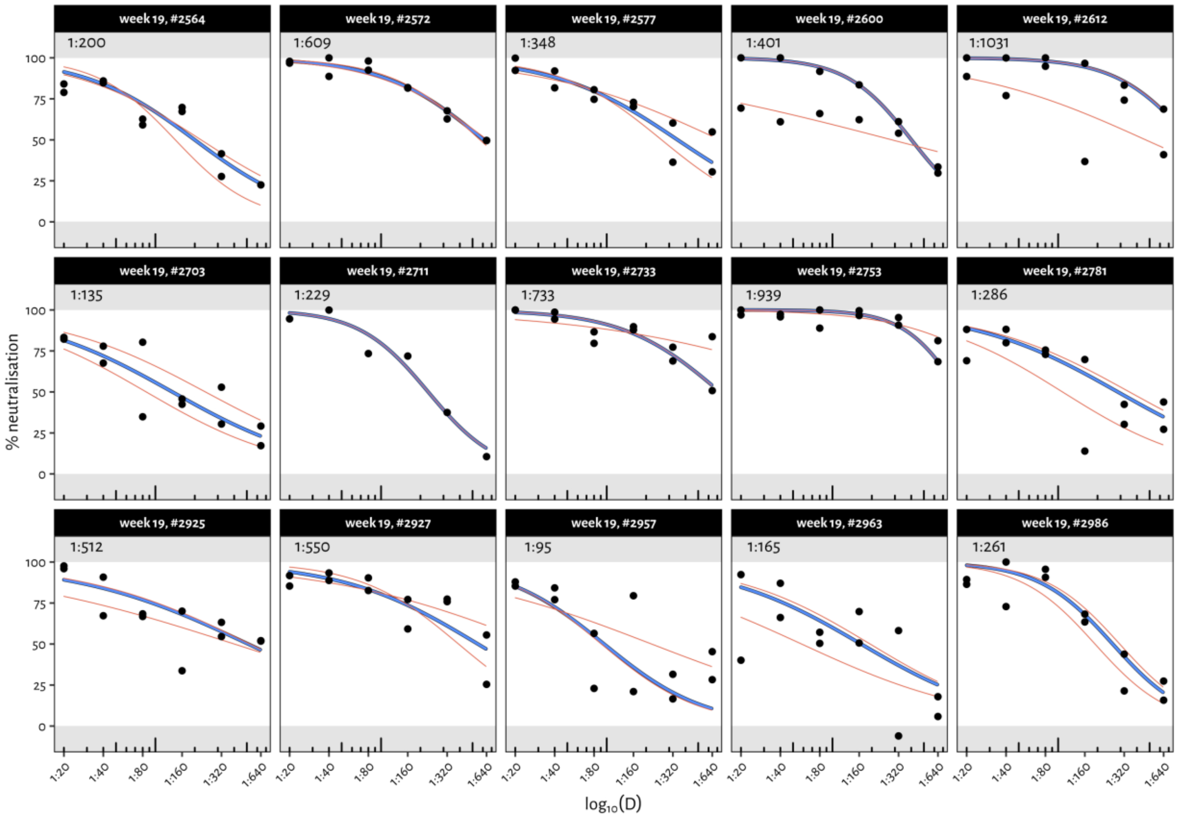

Figure S9. Dilution curves for neutralising samples from week 19. Fits for each replicate are shown in red, with the weighted mean given in blue.

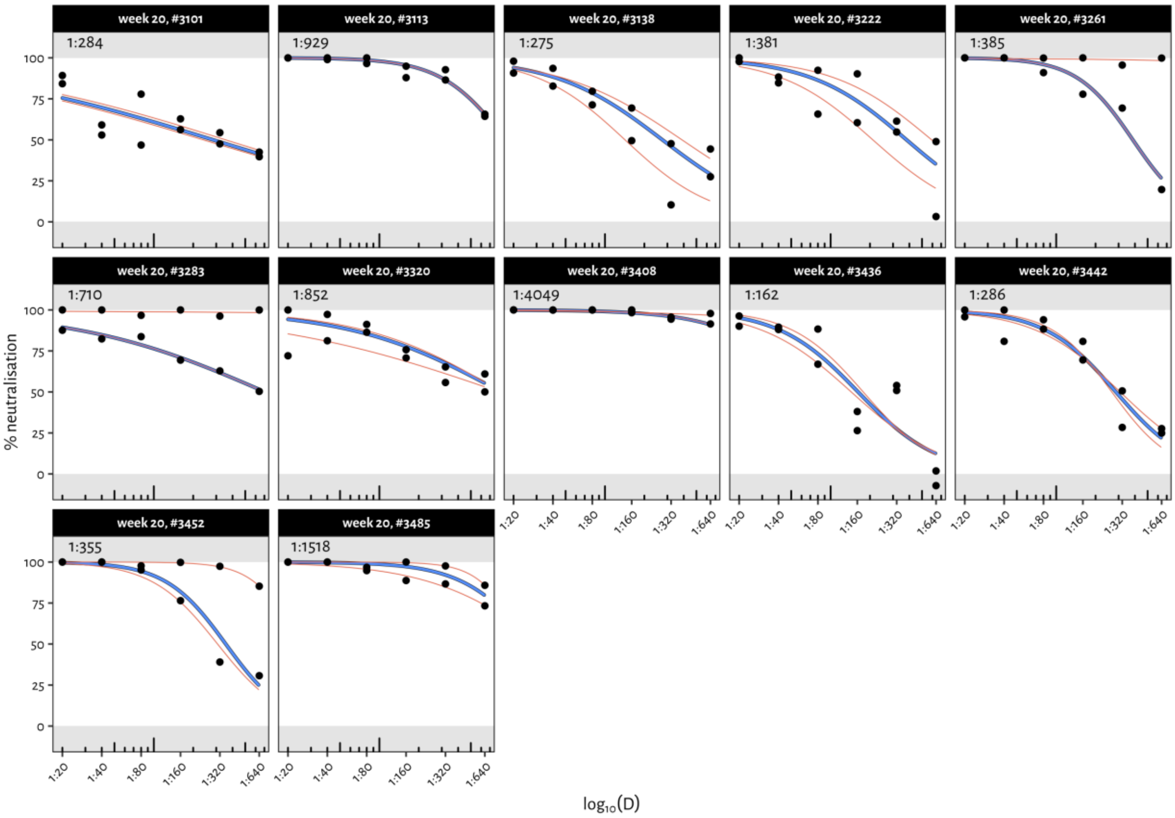

Figure S10. Dilution curves for neutralising samples from week 20. Fits for each replicate are shown in red, with the weighted mean given in blue.
